# Molecular Dynamics Analysis Reveals Structural Insights into Mechanism of Nicotine *N*-Demethylation Catalyzed by Tobacco Cytochrome P450 Mono-Oxygenase

**DOI:** 10.1371/journal.pone.0023342

**Published:** 2011-08-16

**Authors:** Shan Wang, Shuo Yang, Baiyi An, Shichen Wang, Yuejia Yin, Yang Lu, Ying Xu, Dongyun Hao

**Affiliations:** 1 Biotechnology Research Centre, Jilin Academy of Agricultural Sciences, Changchun, China; 2 Key Lab for Molecular Enzymology and Engineering of the Ministry of Education, Jilin University, Changchun, China; 3 Computational Systems Biology Laboratory, Department of Biochemistry and Molecular Biology, and Institute of Bioinformatics, University of Georgia, Athens, Georgia, United States of America; 4 College of Computer Science and Technology, Jilin University, Changchun, China; Massachusetts Institute of Technology, United States of America

## Abstract

CYP82E4, a cytochrome P450 monooxygenase, has nicotine *N*-demethylase (NND) activity, which mediates the bioconversion of nicotine into nornicotine in senescing tobacco leaves. Nornicotine is a precursor of the carcinogen, tobacco-specific nitrosamine. CYP82E3 is an ortholog of CYP82E4 with 95% sequence identity, but it lacks NND activity. A recent site-directed mutagenesis study revealed that a single amino acid substitution, i.e., cysteine to tryptophan at the 330 position in the middle of protein, restores the NND activity of CYP82E3 entirely. However, the same amino acid change caused the loss of the NND activity of CYP82E4. To determine the mechanism of the functional turnover of the two molecules, four 3D structures, i.e., the two molecules and their corresponding cys–trp mutants were modeled. The resulting structures exhibited that the mutation site is far from the active site, which suggests that no direct interaction occurs between the two sites. Simulation studies in different biological scenarios revealed that the mutation introduces a conformation drift with the largest change at the F-G loop. The dynamics trajectories analysis using principal component analysis and covariance analysis suggests that the single amino acid change causes the opening and closing of the transfer channels of the substrates, products, and water by altering the motion of the F-G and B-C loops. The motion of helix I is also correlated with the motion of both the F-G loop and the B-C loop and; the single amino acid mutation resulted in the curvature of helix I. These results suggest that the single amino acid mutation outside the active site region may have indirectly mediated the flexibility of the F-G and B-C loops through helix I, causing a functional turnover of the P450 monooxygenase.

## Introduction

The cytochrome P450 (P450) superfamily of monooxygenases have been identified in all forms of life, i.e., in animals, plants, fungi, protists, bacteria, archaea, and even viruses [1], [2]. P450 plays a major role in drug metabolism and bio-activation, accounting for about 75% of all metabolic reactions. CYP82E4, a member of the CYP82E2 gene family of P450, has NND activity, which mediates the bioconversion of nicotine to nornicotine in senescing tobacco leaves [3]. Nornicotine is a biochemical precursor of the tobacco-specific nitrosamine called *N*′-nitrosonornicotine, which is reportedly carcinogenic to laboratory animals [4]–[6]. In a study on NND-related genes, two closely related genes of CYP82E2 and CYP82E3 were also amplified [3]. CYP82E3 is an ortholog of CYP82E4, with 95% sequence identity at the amino acid level, but it loses NND activity when expressed in yeast and tobacco [3], [7]. Interestingly, a recent site-directed mutagenesis study discovered that the same amino acid substitution causes the functional turnover of CYP82E3 and CYP82E4 [7]; the substitution is Cys330Trp (C330W) in CYP82E3, which corresponds to Trp329Cys (W329C) in CYP82E4. Sequence alignments among P450 proteins from different organisms indicated that the conservation of an aromatic amino acid at this position is essential for NND functionality [7]. However, the detailed mechanism of their interaction is still unclear.

In P450 structures, the active site on the distal side of the heme is buried within the protein interior [8], [9]. The substrate and oxygen molecule are transported to the active site where they bind together for catalysis and hydrogen peroxide is generated as a by-product. After the reaction, the products must leave the protein. The substrate, an oxygen molecule, and the catalyzed products are transferred via different pathways. Thus, the pathways for substrate access and product export play a significant role in the catalysis of P450. Although the mechanism of channel opening is not completely clear at present, multiple active site access channels have been identified in the P450 proteins of different species with different functional states [10]–[14]. The movement of two secondary structure elements, the B-C and F-G loops are essential for channel opening, which border a few channels and act as hinged lids on the conformations of channels [15]. Recent studies have suggested that electron transfer partner protein binding influences the global motion of P450 by changing the motion in the F-G loop region [16]. In addition, increasing the flexibility or variation of length of the B-C loop also affects the opening of relative channels [17], [18]. However, the detailed regulation mechanism of the F-G and B-C loops is unclear.

**Figure 1:**
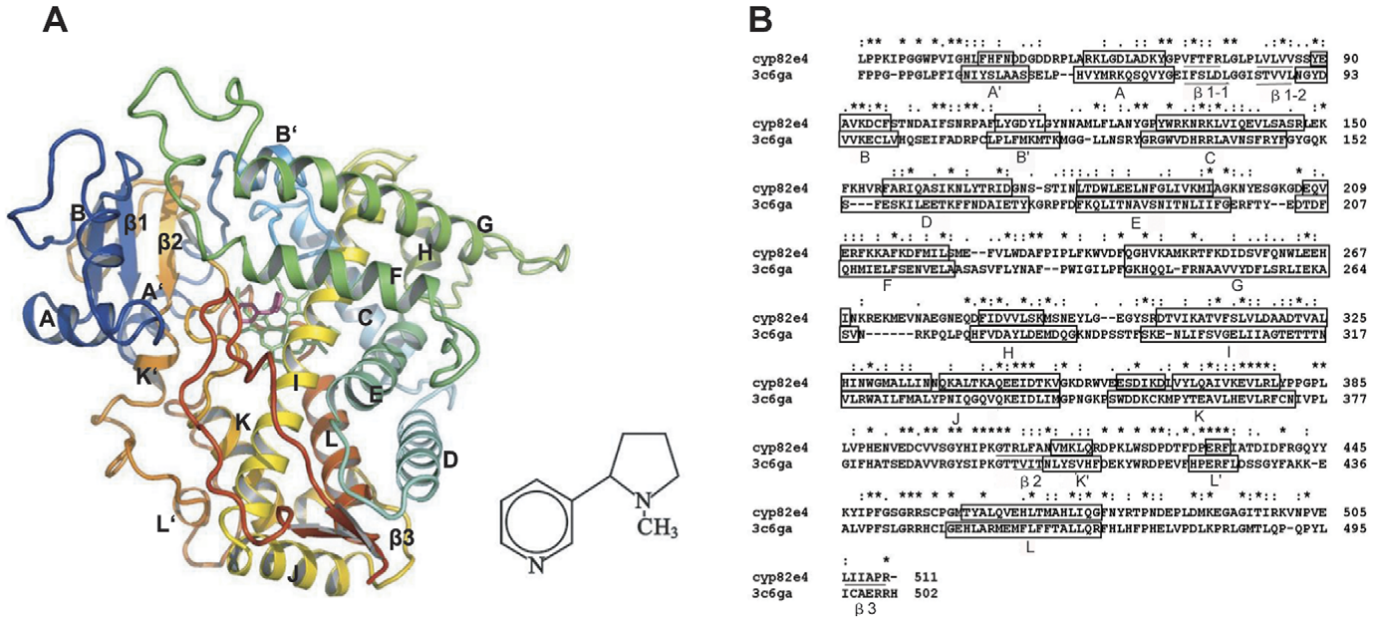
Homology model of CYP82E4 in complex with nicotine and the sequence alignment between CYP82E4 and CYP2R1. (A) Ribbon representation of the CYP82E4 model bound with nicotine. The heme is represented by a green stick; nicotine is represented by a pink stick. Major helices and sheets are labeled. The images were generated using PyMOL (Delano WL (2004); The PyMOL Molecular Graphics System (Delano Scientific LLC, San Carlos, CA, http://www.pymol.org). (B) The sequences were aligned between residues 33 to 511 of CYP82E4 and residues 39 to 503 of CYP2R1. The asterisk indicates an identical or conserved residue, a colon indicates a conserved substitution, and a dot indicates a semi conserved substitution. Boxes and underlines represent helices and β sheets, respectively.

The enzymes CYP82E4 and CYP82E3, which have high sequence identity but different activities, provide a good example for studying the mediation mechanism of complex channel systems. Thus, four distinct homology models for CYP82E4 (Figure 1), CYP82E3, and their cys–trp mutants were constructed to gain a structural insight into their functional mechanism. Six separate molecular dynamics (MD) simulations were performed on CYP82E4, CYP82E3, and their mutants at 300 K, as well as on the wild-type and the mutant for CYP82E4 at 330 K, which improves the reliability of the study. The conformational behaviors of these proteins in both the active and inactive state are analyzed. The transfer channels of CYP82E4 and CYP82E3 are detected in two distinct states. Interestingly, analysis of the MD simulation results suggests that helix I may mediate the flexibility of the F-G and B-C loops. This study provides new insight into understanding the functional mechanism of P450 proteins.

## Results and Discussion

### Mutational effects on the active site

**Figure 2:**
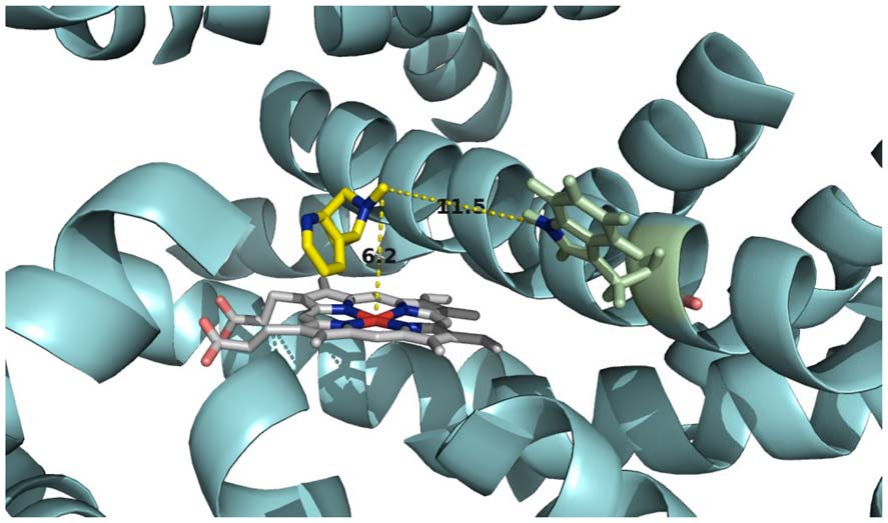
Location of nicotine at active site of CYP82E4. The heme (white), residues Trp329 (green), and nicotine (yellow) are represented as sticks. All helices and sheets are shown as cartoons (cyan). Oxygen, nitrogen and iron atoms are colored salmon, blue and red, respectively. The distance between the iron of heme and methyl group of the nicotine, and the distance between nitrogen of Trp329 and methyl group of nicotine are labeled, respectively.

Four structure models were generated for the wild-type and the mutant CYP82E4 and CYP82E3. The TM-scores for these models are reported in Table 1, where the TM-scores for the predicted and template structures are all close to 0.5, indicating that their structural qualities are acceptable (see Methods for details). The four predicted structures with nicotine (information for docking was provided in table 1) reveal that nicotine is located above the heme plane and near helix I. The distance between the methyl group of nicotine and the Fe of heme is 6.2 Å, whereas the nitrogen of the mutant residue Trp329 in helix I is 11.5 Å from the methyl group of the nicotine in CYP82E4 (Figure 2). Clearly, the mutant residue Trp329 is far from the active site of CYP82E4 and it does not directly interact with the nicotine. This suggests that the mutation does not directly influence the binding of nicotine. Hence, the W329C mutation might have influenced the global conformation rather than the binding with nicotine. Notably, helix I, where the mutant residue Trp329 is located, is part of the substrate-transfer channel of P450 proteins [13], [42], [43].

**Table 1:** Overview of the docking information and assessment of the docked models.

Model	Free-binding energy with heme (kcal/mol)	Ref RMS of heme (Å)	Free binding energy with nicotine (kcal/mol)	Ref RMS of nicotine (Å)	TM-score
Wild-type CYP82E4	−4.79	2.25	−5.77	132.61	0.46490
W329C CYP82E4	−6.48	52.36	−5.69	57.09	0.96391
Wild-type CYP82E3	−9.47	49.87	−6.34	92.12	0.45070
C330W CYP82E3	−9.54	50.54	−6.36	0.14	0.98766

### Global motion

**Figure 3:**
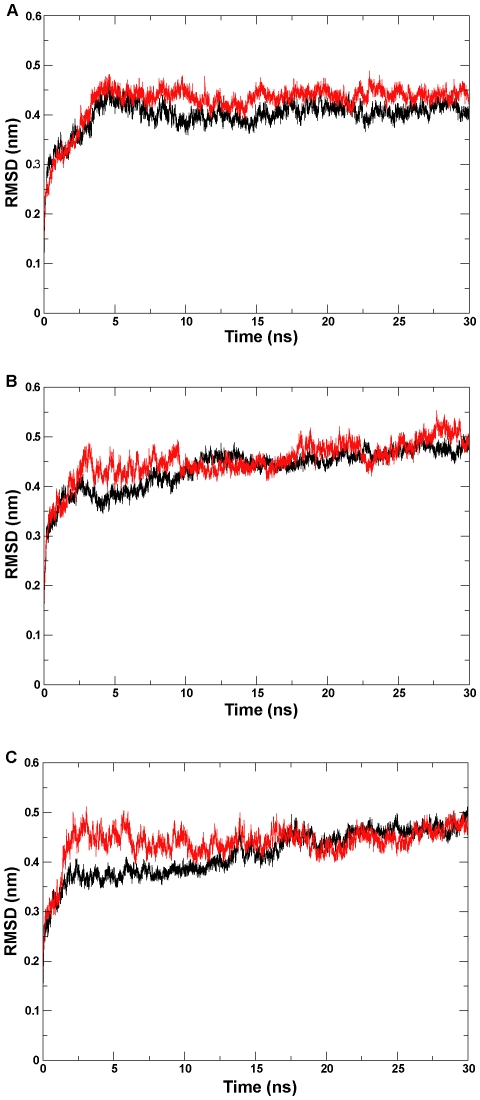
RMSD (Root Mean Square Deviation) profile. The backbone atom RMSD calculated for (A) the wild-type and mutant CYP82E4 at 300 K, (B) the wild-type and mutant CYP82E4 at 330 K, and (C) the wild-type and mutant CYP82E3 at 300 K. The wild-types and mutants for three pairs of ensembles are marked separately with black and red lines.

To assess the conformational stability of the proteins during the MD simulation, the time-dependent the root-mean-square deviation (RMSD) of the backbone atoms with respect to the corresponding initial structures was calculated separately for six simulation systems. As shown in the RMSD profile (Figures 3A, 3B, and 3C), our six simulation systems are reproducible and plateaued during the 30-ns simulation time scale. The final RMSDs were from 0.40 nm to 0.51 nm for the six simulation systems (Table 2).

**Table 2:** Summary of RMSDs for six ensembles.

Simulation name	Time of convergence (ns)	Converged RMSD (nm)
Wild-type CYP82E4 (300 K)	4.8	0.40
W329C CYP82E4 (300 K)	4.8	0.43
Wild-type CYP82E4 (330 K)	12.0	0.48
W329C CYP82E4 (330 K)	3.0	0.51
Wild-type CYP82E3 (300 K)	17.5	0.51
C330W CYP82E3 (300 K)	2.5	0.48

**Figure 4:**
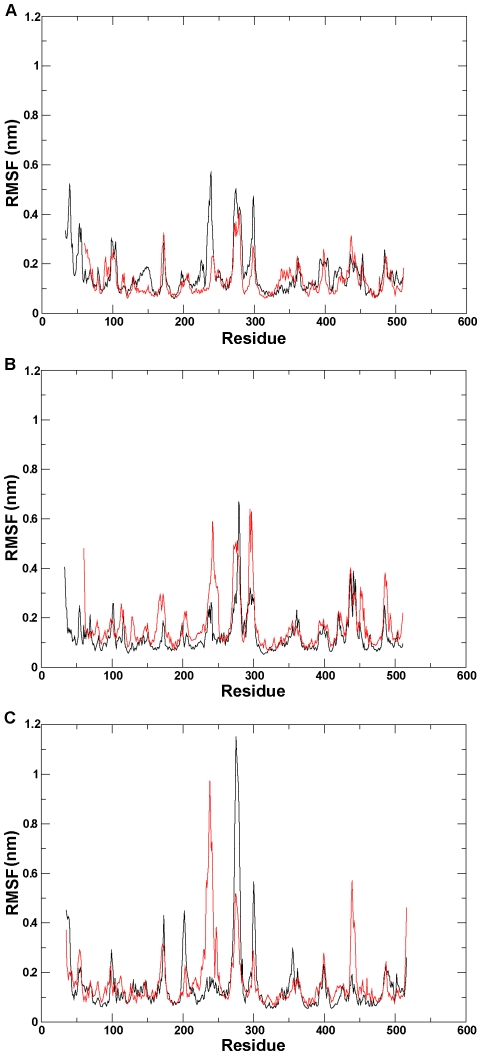
RMSF (Root Mean Square Fluctuation) profile. The C-alpha RMSF per residues calculated for (A) the wild-type and mutant CYP82E4 at 300 K, (B) the wild-type and mutant CYP82E4 at 330 K, and (C) the wild-type and mutant for CYP82E3 at 300 K. The wild-types and mutants for three pairs of ensembles are marked using black and red lines, respectively.

To study the fluctuation of individual residues in detail, the root-mean-square position fluctuation (RMSF) of C-alpha atoms was analyzed for each of the six ensembles (Figure 4). A trimodal distribution in the central region is observed for each of the six RMSF profiles, of which the first one is the region with the largest difference between the mutant and the wild type. This region is located in the F-G loop (from F226 to R251), whose flexibility is crucial for regulation of the opening/closing of P450 channels [15]. The RMSF profiles indicate that the conformational rearrangement took place in the mutants, of which the largest change occurred in the F-G region.

### Principal component analysis

**Figure 5:**
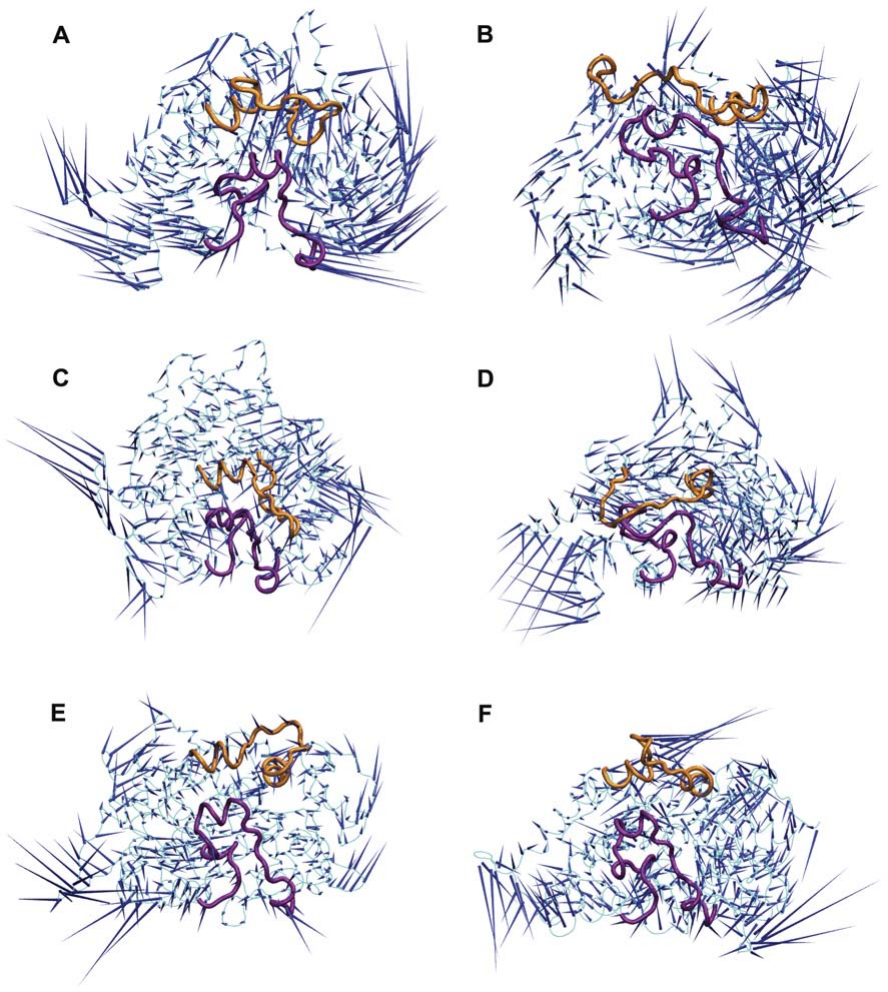
Dominant motions in six simulation ensembles using Principal Component Analysis. Porcupine plot of the first eigenvector in (A) the wild-type CYP82E4 at 300 K, (B) the mutant CYP82E4 at 300 K, (C) the wild-type CYP82E4 at 330 K, (D) the mutant CYP82E4 at 330 K, (E) the wild-type CYP82E3 at 300 K and (F) the mutant CYP82E3 at 300 K. The F-G and B-C loops are marked in yellow and purple, respectively.

The first prominent characteristic motions (PC1) during the simulation were analyzed through principal component analysis (PCA). The top eigenvectors that correspond to the PC1 accounted for 60%, 59%, 62%, 51%, 62%, and 54% of the motions in the wild-type and mutant for CYP82E4 at 300 K, the wild-type and mutant for CYP82E4 at 330K, and the wild-type and mutant for CYP82E3 at 300 K, respectively. Our observation focused on the movements of the F-G and B-C loops. We found that the flexible motions of these two secondary structures in the distinct active states are quite different (Figure 5). Specifically, the F-G loop moves downwards or upwards in the wild-type and the mutant for CYP82E4 at 300 K, respectively; moves outwards or inwards, respectively in the wild-type and the mutant for CYP82E4 at 330 K; and moves leftwards or rightwards, respectively in the wild-type and mutant for CYP82E3 at 300 K. In contrast, the B-C loop has an expanding or contracting motion in the wild-type and mutant for CYP82E4, respectively, at both 300 K and 330 K, which is the opposite in the wild-type and mutant for CYP82E3 at 300 K. A similar observation was made on CYP2C5 [17]. Overall, the mutation results in a change in the motion patterns of these two secondary elements.

### Covariance analysis

**Figure 6:**
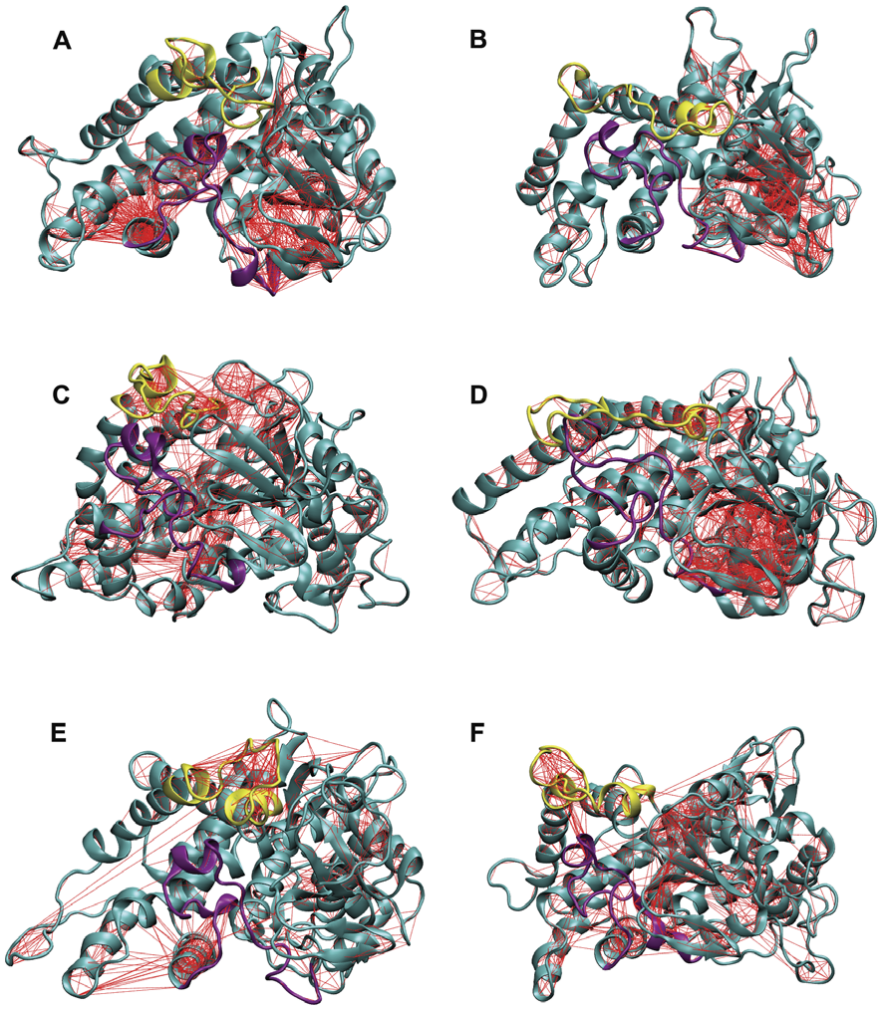
3D structure plot with covariance lines. The pair-wise covariance for C-alpha atoms in a 3D representation for the six simulation ensembles. The correlation coefficients of the pair-wise atoms >70% are considered as having correlated motions. (A) the wild-type CYP82E4 at 300 K, (B) the mutant CYP82E4 at 300 K, (C) the wild-type CYP82E4 at 330 K, (D) the mutant CYP82E4 at 330 K, (E) the wild-type CYP82E3 at 300 K, and (F) the mutant CYP82E3 at 300 K. The F-G and B-C loops are marked in yellow and purple, respectively.

To understand better the correlation between the motions of the F-G and B-C loops, as well as the motions of the surrounding region, covariance analyses were performed on the six ensembles and they were visualized using 3D structure plots. We observed a mass with correlated motions between the two secondary structures and the surrounding region in the active state of the relevant ensembles. The correlated motions of these regions decreased considerably in the inactive state (Figure 6), which indicates that the synchronization of these regions receded and suggests that the flexibility of the F-G and B-C loops is responsible for the motions of the surrounding pathways. This is consistent with the viewpoint of a previous study [15]. In addition, the motions of these two secondary structures are correlated with the motion of helix I, which is important for the conformational stability of P450 [44], [45]. In the following sections, we detected cavities in the distinct conformations over the plateaued dynamics trajectory for six simulation systems. A more direct analysis is shown to prove the detailed regulatory relationship between the motions of the B-C and F-G loops and the motions of the surrounding channels.

### Channel analysis

**Figure 7:**
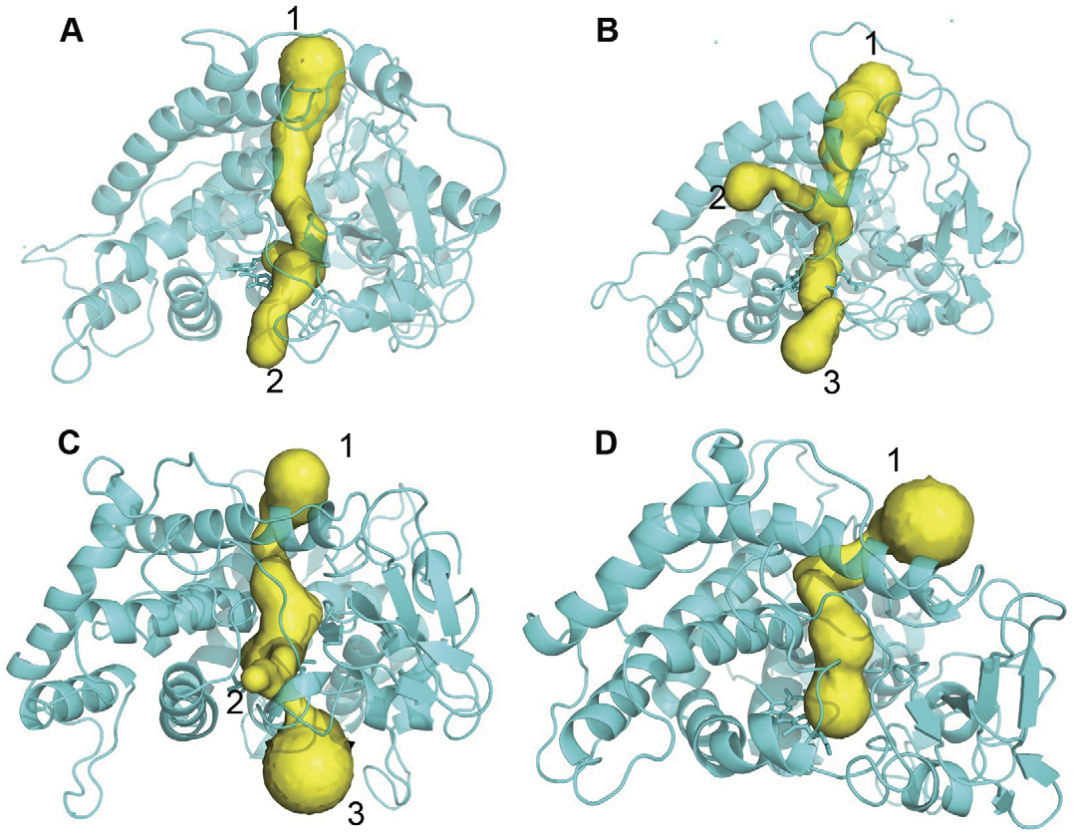
Channel analysis of CYP82E4 at 300 K. The channels were detected in the wild-type CYP82E4 at 300 K in (A) closing mode, which corresponds to the conformation at 16 ns, and (B) opening mode, which corresponds to the conformation at 29 ns, and in the mutant CYP82E4 at 300 K in (C) opening mode, which corresponds to the conformation at 10 ns and (D) closing mode, which corresponds to the conformation at 24.5 ns.

**Figure 8:**
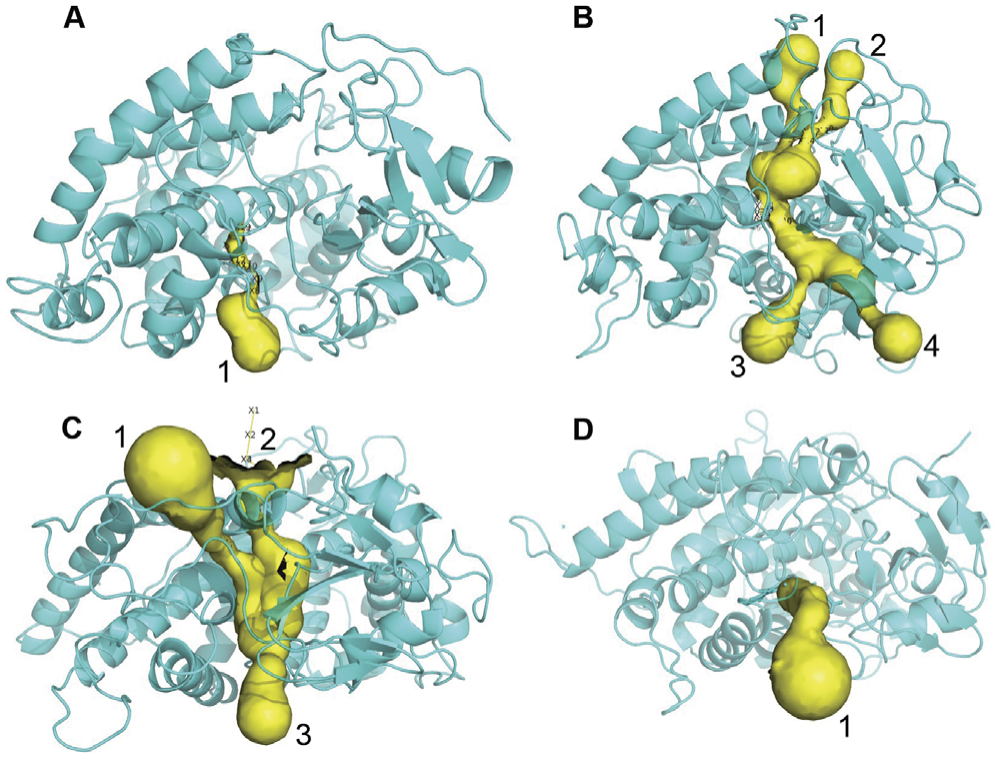
Channel analysis of CYP82E4 at 330 K. The channels were detected in the wild-type CYP82E4 at 330 K in (A) closing mode, which corresponds to the conformation at 12.5 ns and (B) opening mode, which corresponds to the conformation at 20 ns, and in the mutant CYP82E4 at 330 K in (C) opening mode, which corresponds to the conformation at 17 ns and (D) closing mode, which corresponds to the conformation at 30 ns.

**Figure 9:**
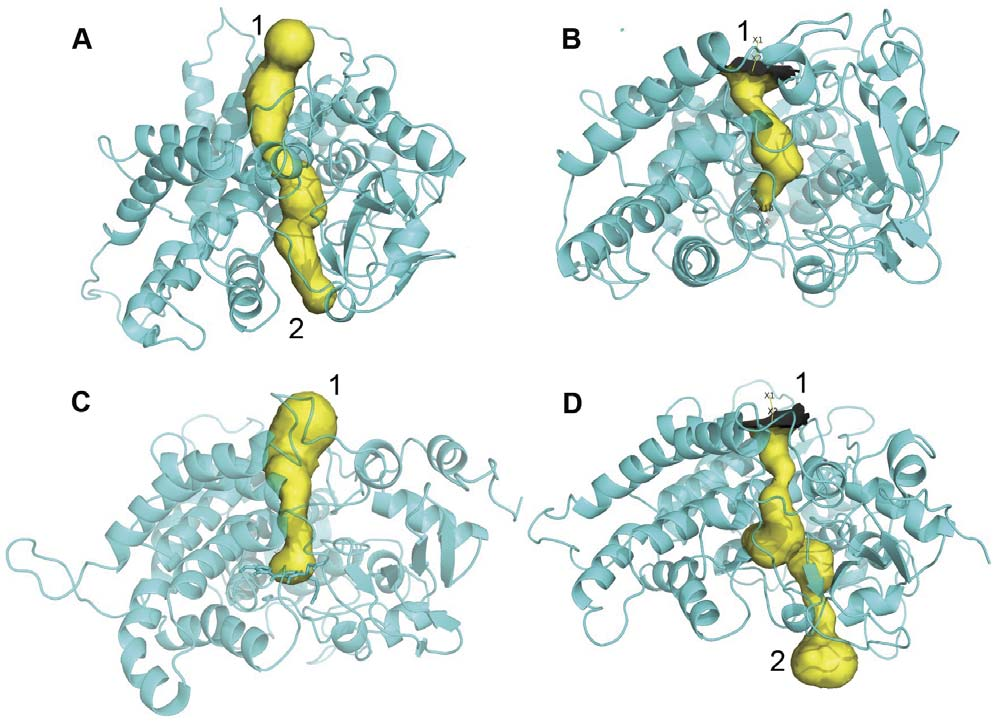
Channel analysis of CYP82E3 at 300 K. The channels were detected in the wild-type CYP82E3 at 300 K in (A) opening mode, which corresponds to the conformation at 17.5 ns, in (B) closing mode, which corresponds to the conformation at 30 ns, and in the mutant CYP82E3 at 300 K in (C) closing mode, which corresponds to the conformation at 5 ns, and (D) opening mode, which corresponds to the conformation at 17.5 ns.

To determine the associated motion between the F-G and B-C loops and the surrounding channels, the channels with distinct conformations over the simulation stages were detected in six simulation ensembles. Specifically, based on the PCA analysis, both the F-G loop and the B-C loop of the wild-types and mutants move in opposite directions. Thus, the distances between the F-G and B-C loops were analyzed across the whole simulation trajectory for six simulation ensembles (Figure S1). The conformations corresponding to the two extreme distances between two loops during the stable simulation phases were extracted from the simulation trajectory for each of the six simulation ensembles. The channels in the extreme conformations were identified using CAVER. The number of channels in the active conformations tends to increase, which indicates the opening of the channel, as shown in Figures 7A, 7B, 8A, 8B, 9C, and 9D. In contrast, the channels in the inactive conformations show a decline in the number, as shown in Figures 7C, 7D, 8C, 8D, 9A, and 9B. In addition, only one channel remains in the inactive state, which corresponds either to a pathway for substrate access/product egress (Figures 7D and 9B) or to a pathway for water egress (Figure 8D) [15]. Overall, this suggests that the monooxygenase reaction of P450 requires that the pathways for substrate access, product egress, and water egress to be open coordinately, which is consistent with the P450 mechanism mentioned in the introduction. It also shows that the mutation affects the channel opening-closing movement by altering the motion of the F-G and B-C loops. However, a question arises regarding how the mutation site affects the motion of the F-G and B-C loops.

### Analysis of properties of helix I

**Figure 10:**
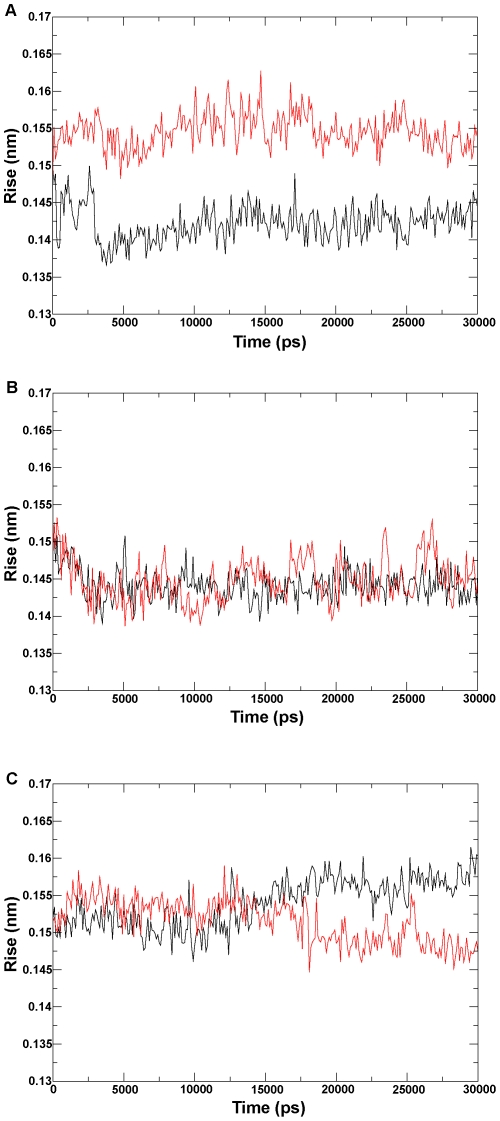
Analysis of helical rise per residue. The helical rise per residue is analyzed for (A) the wild-type and mutant CYP82E4 at 300 K, (B) the wild-type and mutant CYP82E4 at 330 K, and (C) the wild-type and mutant CYP82E3 at 300 K. The wild-types and mutants for the three pairs of ensembles are marked using black and red lines, respectively.

**Figure 11:**
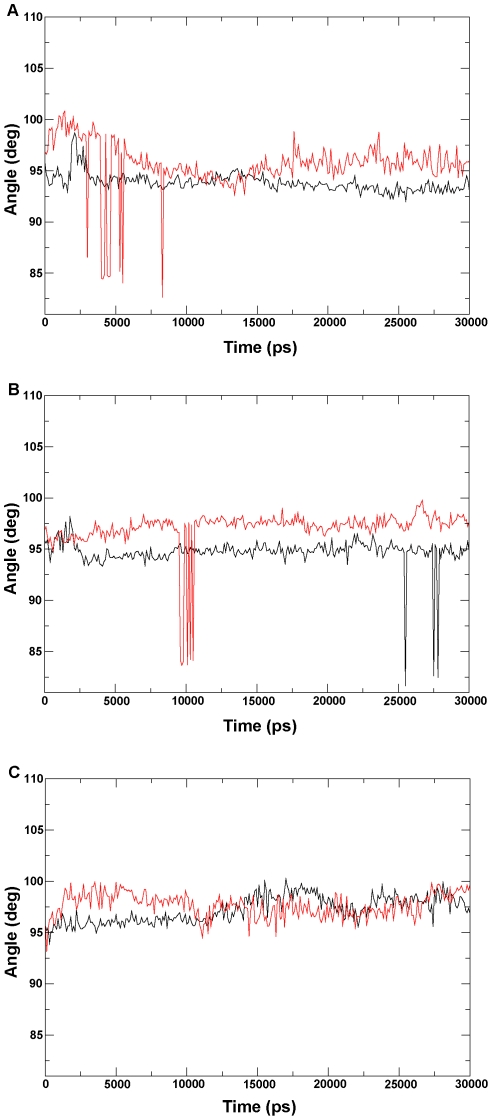
Twist analysis of helix I. The helical twist is analyzed for (A) the wild-type and mutant CYP82E4 at 300 K, (B) the wild-type and mutant for CYP82E4 at 330 K, and (C) wild-type and mutant for CYP82E3 at 300 K. The wild-types and mutants for the three pairs of ensembles are marked using black and red lines, respectively.

A previous study suggested that the *N*-terminus of helix I plays a role in equilibrium between the opening and closing conformations of P450 enzymes [45]. The I290F mutation at this position in P450 2B11 reduces the benzphetamine-binding affinity, which has a *Ks* 20% of that of the wild-type enzyme [46]. To detect the effect of the mutation on the helix I of P450 proteins, we concentrated on the curl of helix I. The helical rise per residue and the helical twist were measured over the whole dynamics trajectory for six ensembles (see Methods for details). In the two 300 K systems (Figures 10A and 10C), the helical rise per residue in the active states (the wild-type CYP82E4 and the C330W CYP82E3) is smaller than that in the inactive states (the W329C CYP82E4 and the wild-type CYP82E3). In the 330 K systems (Figure 10B), the differences in helical rise per residue were not significant in the two states. In addition, the degrees of the helical twist in the active state for the two CYP82E4 systems (Figures 11A and 11B) were smaller than that in the inactive states. For the CYP82E3 systems (Figure 11C), the curves for degrees of the helical twist intersected in two states, but were always complementary. Overall, our analysis of helix I suggests that helix I during the inactive state of P450 proteins tends to be curled, which affects the motion of the F-G and B-C loops, as revealed by the covariance analysis.

### Conclusions

Six MD simulations, totaling 180 nanoseconds in time-scale, were performed to study the conformational behaviors of CYP82E4 and CYP82E3 in different biological scenarios. The dynamics trajectories analyses using PCA and the basic properties analysis of helix I suggest that the mutation alters the motion of the F-G and B-C loops through the curling of helix I. The correlated motions were observed between the regions of both the F-G and B-C loops and the surrounding regions, which suggest that the F-G and B-C loops, as the hinges of multiple channels, regulate channels opening and closing. Hence, the mutation site indirectly affects the opening and closing of the relevant channels, which is also supported by the analysis of the channels in the conformations of the six simulation systems. In addition, the analysis of the channel in the distinct active conformations suggests that the monooxygenase activity of CYP82E4 and CYP82E3 require the pathways for substrate access, products egress, and water egress to be open coordinately. The current study explains the enzymatic mechanisms of CYP82E4 and CYP82E3, which are mediated by a single amino acid in helix I outside the active site region.

## Methods

### Structures preparation

P450 monomers are known to have activity, although P450 proteins generally function as dimers. Hence, only the monomer structures of CYP82E4 and CYP82E3 were modeled. The sequences of CYP82E4 (gi: 121053143) and CYP82E3 (gi: 74475192) were obtained from the Protein Database of NCBI. Two mutational sequences were obtained by replacing Trp329 with Cys329 in the sequence of CYP82E4 and by replacing Cys330 with Trp330 in the sequence of CYP82E3. Based on these protein sequences, four 3D structural models were constructed using the homology modeling program SWISS-MODEL [19]–[21]. The templates were selected based on high sequence identity. Specifically, 1) a structural model for CYP82E4 (only considered residues 33–511) was constructed using the crystal structure of subunit A of CYP2R1 complexed with vitamin D3 (PDB entry 3C6G) [22] as the template; 2) the W329C mutant of CYP82E4 (residues 60–511) was constructed using the crystal structure of subunit C of P450 2D6 (PDB entry 2F9Q) [23] as the template; and 3) CYP82E3 and the C330W mutant (residues 35–516) were constructed separately using the crystal structure of subunit B of CYP2R1 complexed with vitamin D2 (PDB entry 3CZH) as the template. The structural quality of the predicted models were assessed using TM-score [24], [25], the currently most popular method for assessing structural qualities. The value of the TM-score lies within (0, 1), with a TM-score<0.17 indicating there is no similarity between the two structures under consideration; and a TM-score>0.5 indicating that the two structures share the same structural fold.

Heme and nicotine were separately docked into the active sites of the aforementioned four models using the grid-based docking program AUTODOCK 4.2.2.1 [26], with a modified genetic search algorithm plus a local minimum refinement procedure [27]. The structure of heme was extracted from the crystal structure of subunit A of CYP2R1 complexed with vitamin D3.

### Molecular dynamics simulations

To study the enzymatic activity of CYP82E4 and CYP82E3 in details, we prepared the conformational ensembles of the wild-type and mutant CYP82E4 and CYP82E3 over the course of the MD simulation at an initial temperature of 300 K, as well as those of the wild-type and mutant CYP82E4 at 330 K matched with those at 300 K. The GROMACS program (version 4.5.3) [28]–[30] with the GROMOS96 53a6 force field [31], [32] was used to perform MD simulations on six models; their functional states are outlined in Table 3. Each system was solvated in a dodecahedron periodic box with a simple point charge (SPC) water model [33]. The distance between the solute and the box was 10 Å for each system, i.e., having a layer of water at least 10 Å thick between each protein and the boundaries of the dodecahedron box. The topology file of nicotine for GROMOS96 force field was generated using the Dundee PRODRG Server [34]. The added Na+ ions for neutralizing the box charge and the total atoms of each ensemble are shown in Table 3. The structure of each ensemble was relaxed through 50,000 steps of energy minimization. Equilibration of the ensembles was conducted in two phases: the phase of 100 ps constant NVT (Number of particles, Volume, and Temperature) simulation, and the phase of 100 ps constant NPT (Number of particles, Pressure, and Temperature) simulation. Afterward, a 30 ns MD simulation was conducted on each ensemble. The initial temperature is given in Table 3. The thermostat and barostat coupling on each ensemble was done separately using the Nose–Hoover and Parrinello–Rahman methods. The electrostatic interactions were calculated using the particle-mesh Ewald method [35] with a 1.0 nm cutoff. During MD simulation, the LINCS algorithm [36] was used to constrain all the bonds.

**Table 3:** Simulation parameters for six systems.

System	Number of NA^+^ ions	State	Temperature (K)	Number of atoms
Wild-type CYP82E4	5	Active	300	18,562
W329C CYP82E4	3	Inactive	300	16,268
Wild-type CYP82E4	5	Active	330	18,562
W329C CYP82E4	3	Inactive	330	16,268
Wild-type CYP82E3	2	Inactive	300	18,228
C330W CYP82E3	0	Active	300	18,230

### RMSD, RMSF, Helix, and Channel analysis

To compare the global structural variations during the simulation, RMSDs for the backbone atoms of six simulation ensembles were calculated with respect to the corresponding initial structure of each simulation trajectory as a function of time. The C-alpha RMSFs were measured to detect the positions of the individual atoms with respect to the average position across the whole simulation trajectory for six ensembles.

The basic properties of helix I, the helical rise per residue and the helical twist, were analyzed using the g_helix program in GROMACS to observe the mutational effect on the curl of helix I in the MD simulations. Specifically, the helical rise per residue is plotted as the difference in Z-coordinate between Ca atoms. The helical twist is described by the average helical angle per residue.

To compare the mutational effects on pathways going from the buried cavities to the outside solvent, the conformational variations of each channel during the MD simulation were detected using CAVER [37] on protein structures for each of the six simulation ensembles.

### Principal component analysis and covariance analysis

To identify the most prominent characteristics of the motions along a simulation trajectory, PCA [38], [39] was used to detect the direction and amplitude of the dominant motions. Specifically, the Dynatraj v1.5 program [40] was used to perform PCA, which generates a *porcupine plot* showing a graphical summary of the motions along the trajectory. In a porcupine plot, each C-alpha atom has a cone pointing in the direction of the motion of the atom; the length of the cone reflects the amplitude of the motion and the size of the cone indicates the number of such C-alpha atoms. The pair-wise covariance for C-alpha atoms was detected through covariance analysis [41], which was represented in a 3D structure plot using the Dynatraj v1.5 program. The covariance value ranges from −1 to +1, where, +1 value indicates that the corresponding pair of atoms moves together at all times; −1 for pairs of atoms moving in opposite directions at all times; and 0 means that the motions of the two atoms are uncorrelated. In the 3D structure plot, the covariance lines were drawn between the atoms with covariance value >0.7.

## Supporting Information

Figure S1 **Distance analysis between the F-G loop and the B-C loop.** The distance between the centers of mass of the F-G and B-C loops is calculated as a function of time for (A) the wild-type CYP82E4 at 300 K, (B) the mutant CYP82E4 at 300 K, (C) the wild-type CYP82E4 at 330 K, (D) the mutant CYP82E4 at 330 K, (E) the wild-type CYP82E3 at 300 K, and (F) the mutant CYP82E3 at 300 K. The largest and the shortest distances between the two loops during the stable simulation phases are marked by red lines.
(TIF)
