# Supplementary figures and images for: Molecular Dynamics Analysis Reveals Structural Insights into Mechanism of Nicotine N-Demethylation Catalyzed by Tobacco Cytochrome P450 Mono-Oxygenase
Source: PLoS One. 2011 Aug 16;6(8):e23342. doi: 10.1371/journal.pone.0023342 (PMC3156719; doi:10.1371/journal.pone.0023342)

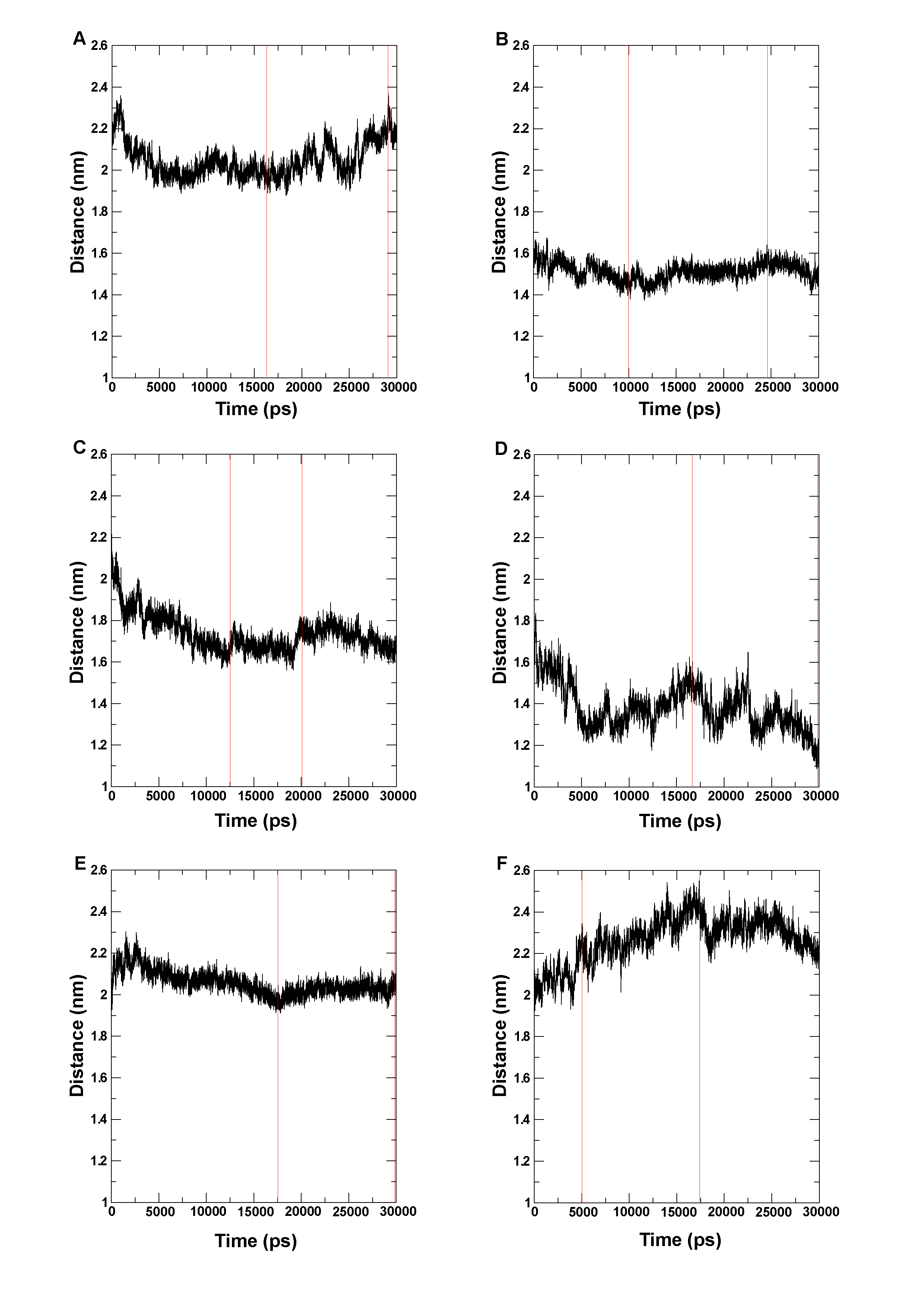

Supplement: Figure S1 — Distance analysis between the F-G loop and the B-C loop. The distance between the centers of mass of the F-G and B-C loops is calculated as a function of time for (A) the wild-type CYP82E4 at 300 K, (B) the mutant CYP82E4 at 300 K, (C) the wild-type CYP82E4 at 330 K, (D) the mutant CYP82E4 at 330 K, (E) the wild-type CYP82E3 at 300 K, and (F) the mutant CYP82E3 at 300 K. The largest and the shortest distances between the two loops during the stable simulation phases are marked by red lines. (TIF) [file pone.0023342.s001.tif]
